# Aflatoxins contamination in spices marketed in selected areas of Tanzania and their Detection by Chromatographic Technique

**DOI:** 10.4314/ahs.v24i2.17

**Published:** 2024-06

**Authors:** Sharifa Juma, Clarence Mgina, Kessy F Kilulya

**Affiliations:** Chemistry Department, University of Dar es Salaam, P. O. Box, 35061 Dar es Salaam, Tanzania

**Keywords:** Aflatoxins, mycotoxins, liquid chromatography, contamination, spices

## Abstract

**Background:**

Aflatoxins are a family of toxins produced by fungi species known as Aspergillus flavus and Aspergillus parasiticus. Contamination of aflatoxins in agricultural crops is of high concern as it has negative effects on public health.

**Objective:**

This study reports on the levels of aflatoxins in four types of spices (black pepper, turmeric, cardamom and garlic) collected from markets, stores and farms in selected locations of Tanzania.

**Methods:**

A total of 84 samples of selected spices were collected. The determination of aflatoxins was performed using high-performance liquid chromatography, coupled with fluorescence detector.

**Results:**

The results obtained showed that 64 samples (76%) were contaminated with total aflatoxins at varying levels with respect to location and weather conditions. Mean concentrations of total aflatoxins ranged from < DL to 8.41 ngg^-1^ for black pepper, from < DL to 0.22 ngg^-1^ for garlic, from < DL to 11.07 ngg^-1^ for cardamom and from 0.28 to 2.21 ngg^-1^ for turmeric. 4.7% of samples exceeded the maximum tolerable limit of 10 ngg^-1^ for total aflatoxins (TAF) set by European Commission and 7 (8.33 %) samples exceeded the maximum tolerable limit of 5 ngg^-1^ for Aflatoxin B_1_.

**Conclusion:**

The observed aflatoxin contamination gives an alert for control of aflatoxins for improved public health.

## Introduction

Aflatoxins are a family of toxins produced by fungal species that grow on different agricultural crops such as maize, cereal and cereal products, nuts, sunflower, cotton seeds, dry fruits and spices. The main species of fungi that produce aflatoxins are *Aspergillus flavus* and *Aspergillus parasiticus*, which are abundant in warm and humid regions of the world. Over 5 billion people in developing countries worldwide are in danger of prolonged exposure to aflatoxins through contaminated food and feed.^1^, ^2^ The contamination of aflatoxins in different agricultural crops and products is of high concern as it has negative effects to food security and public health. Different types of aflatoxins are known, however, only four types of them; aflatoxins B_1_ (AFB_1_), B_2_ (AFB_2_), G_1_ (AFG_1_) and G_2_ (AFG_2_) have got more attention worldwide due to their reported acute liver effects of human and animals leading to liver cancer. Aflatoxins are among the most potent carcinogenic, teratogenic and mutagenic compounds in nature.^3^,^4^ Aflatoxins also cause nutrient alteration like vitamin A or D in animals thus making them inaccessible for the normal body physiology leading to nutritional insufficiencies.^5^ Aflatoxins obstructs nucleic acid synthesis (DNA-RNA) and interferes with the detoxification process leading to mutation hence liver cancer since the main target is the liver.^6^, ^7^ They are also known to reduce sperm count and cause infertility, low birth weight and child defects (teratogenic) as well as decreasing milk and egg production.^8^ The toxicity of aflatoxins has been reported to be in the order of aflatoxin B_1_ (AFB_1_) > aflatoxin G_1_ (AFG_1_) > aflatoxin B_2_ (AFB_2_) > aflatoxin G_2_ (AFG_2_).^9^

**Figure:**
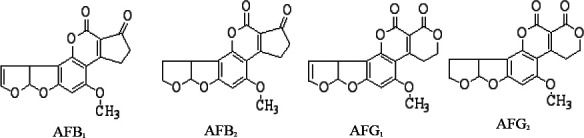


Humans are exposed to aflatoxins by ingestion of contaminated food such as milk and dairy products, cereals, cacao, oilseeds (cotton, groundnuts, sunflower), coffee, grapevine, dried fruit seeds and various spices.^10^, ^11^

Spices are food additives commonly used as flavoring, seasoning, colorant and imparting aroma. Thus, they are used as food cooking additives in Tanzania like in any other country. Different types of spices are grown in several regions in Tanzania including Arusha (garlic), Morogoro (turmeric), Zanzibar (black pepper, turmeric), Tanga (cardamom, black pepper, turmeric), Mbeya (garlic), and some are imported from India. These spices are highly consumed in nearly every household as major additives in food, used as medicines (black pepper, garlic, turmeric) and as natural cosmetics (garlic, turmeric). Spices are contamiated by aflatoxins because of insanitary production process, poor handling and storage conditions. They are highly contaminated by dust and wastewater when left unpacked at different selling levels.^12^ Proper handling, drying, transportation and storage of spices are of paramount importance for eliminating/minimizing aflatoxin contaminations. Among the spices consumed in Tanzania are imported from different countries while some of them are cultivated within the country particularly Zanzibar and Arusha. Farmers and other agricultural workers may be at risk of gasping dust containing aflatoxins created during handling and processing of contaminated crops and feed. Aflatoxins are stable to heat, hence cannot be totally eradicated in agricultural products like spices under normal cooking temperature.^13^

Incidences of aflatoxins contaminations in different types of spices have been reported from different places around the world. For example, in Ethiopia, a research conducted on 60 samples of spices collected from markets, shops and storage facilities revealed that, eight samples were contaminated with Aflatoxin B_1_ in concentrations ranging from 250-525 ngg^-1^ 14, which is far above the maximum set limits. Results of a study conducted in India on aflatoxin contamination in spices, reported up to 120 ngg^-1^ aflatoxins levels in 18 out of 125 samples of black pepper, ginger and turmeric assembled from drying yards of Kerala and warehouse of Karnataka.^15^ Moreover, a study conducted in Nyahururu markets in Kenya by Mwangi et al. 2014 on 13 types of spices (chili, curry powder, cayenne, paprika, cinnamon, pepper, ginger, cloves, garlic, nutmeg, turmeric, cumin and mixed spices) revealed that 50% of the samples had aflatoxins levels beyond the tolerable limits set by EC (10 ngg^-1^).^16^ In Tanzania, several studies on levels of aflatoxins in food like maize, cereals, sunflower and groundnuts have been conducted.^17^-^22^ However, none of these studies reported on the levels of aflatoxins in different spices produced and/or marketed in Tanzania. This paper therefore, reports on the levels of aflatoxins in four types of spices; black pepper, turmeric, cardamom and garlic which are produced and/or marketed in Arusha and Zanzibar.

## Materials and Methods

### Sample Collection

**Figure 1:**
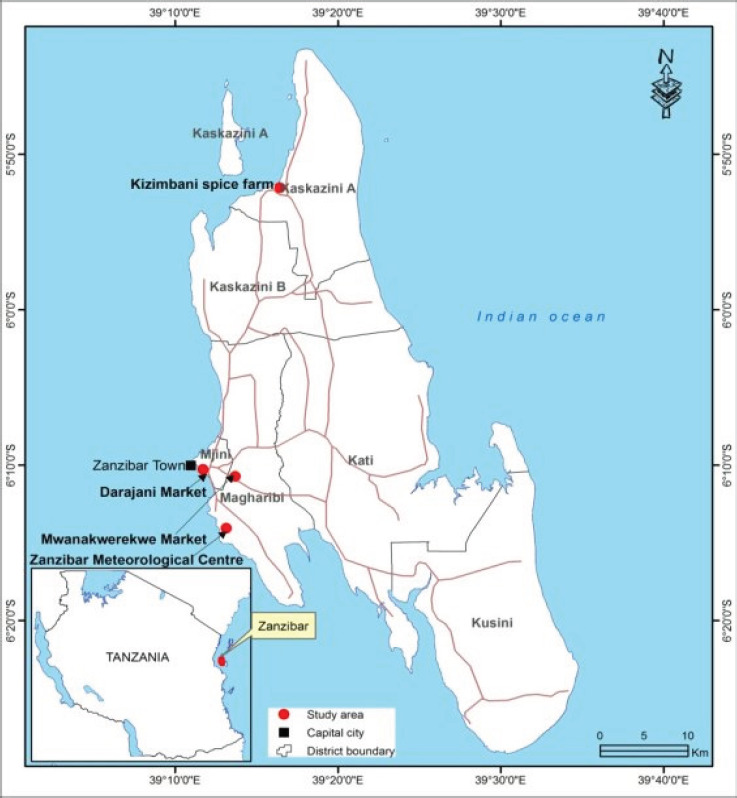
A Map of Zanzibar Showing Sampling Sites

**Figure 2:**
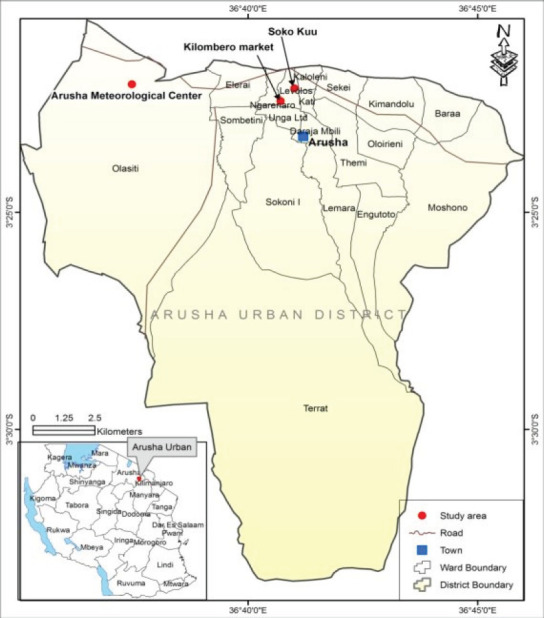
A Map of Arusha Showing Sampling Sites

The samples of selected spices (cardamom, garlic, black pepper and turmeric) were randomly collected from five sampling sites in Zanzibar (Figure 1) and Arusha (Figure 2). In Zanzibar samples were collected from Mwanakwerekwe market and its associated stores, Darajani market and KZ spices farm whereas in Arusha, samples were collected from Kilombero market, stores and Soko Kuu. A total of eighty-four (84) samples (20 cardamom, 20 garlic, 22 turmeric and 22 black pepper) were collected from the two regions (Tables 1 and 2). Collected samples were then packed in polyethylene bags to avoid moisture and transported to the Tanzania Bureau of Standard (TBS) laboratories where they were refrigerated at a temperature of 4 °C, prior to laboratory analyses.

**Table 1:** Samples Collected from Markets and Store in Arusha, Tanzania

Number and Types of Collected Spices Samples					

Sampling sites (Markets and Stores)	Cardamom	Garlic	Turmeric	Black Pepper	Total
SK	3	3	3	3	12
KL	3	3	3	3	12
KLs	3	3	3	3	12
TOTAL	9	9	9	9	36

SK = Soko Kuu Market, KL = Kilombero Market, KLs = Kilombero Stores.

**Table 2:** Samples Collected from Markets, Store and Farms at Zanzibar, Tanzania

Number and Types of Collected Spice Samples					

Sampling Sites (Market, Stores and Farm)	Cardamom	Garlic	Turmeric	Black Pepper	Total
DJ	4	4	4	4	16
MK	4	4	4	4	16
MKs	3	3	3	3	12
KZf	-	-	2	2	4
TOTAL	11	11	13	13	48

DJ = Darajani Market, MK = Mwanakwerekwe Market, MKs= Mwanakwerekwe Stores, KZf = A spice farm in Zanzibar.

### Extraction of Samples

Spice samples; cardamom, garlic, turmeric and black pepper were ground in a blender to get fine particles that could be easily extracted for aflatoxins. In Erlymeyer flask, 25 g of the sample were weighed using analytical balance, and then 2 g of NaOH was added to reduce the oily nature of the samples and hence ensuring optimal extraction. Thereafter 100 mL of extraction solvent (methanol: water (70:30 v/v)) was added into the flask containing the sample. The mouth of the flask was covered with aluminium foil and shaken using gyratory shaker for 30 minutes at 250 rpm followed by filtration using Whatman filter paper (Whatman No.1).

### Dilution and Clean-up

The extract (4 mL) was taken into a Teflon tube followed by addition of 8 mL of double distilled water and vortexed for 30 seconds to ensure adequate mixing. Extracts were cleaned using immunoaffinity columns in which the samples were loaded and allowed to pass through the column by gravity. The columns were washed twice with 10 mL distilled water, subjected to vacuum sucking and thereafter the bounded aflatoxins were eluted with 1 mL of HPLC grade methanol by gravity. Slight pressure was applied to remove any remaining liquid followed by vortexing for 30 seconds. Then, 300 µL of the eluted solution was taken and mixed with 700 µL of the mobile phase (600 µL Water: 100 µL Acetonitrile) and vortexed for 1 minute. Thereafter, the analyte was determined using HPLC-FD based on post-column derivatization.


1 $$\text{HPLC conc}=\frac{\text{Peak}\;\text{Area}\;\text{of}\;\text{the}\;\text{sample}\;(\text{PA})}{\text{Response}\;\text{factor}\;(\text{Rf})}$$

Where,


2 $$\text{Rf}=\frac{\text{Peak}\;\text{area}\;\text{of}\;\text{known}\;\text{concentration}\;\text{of}\;\text{standard}\;\text{solution}}{\text{Known}\;\text{concentration}\;\text{of}\;\text{the}\;\text{standard}\;\text{solution}}$$

3 $$\text{Conc}\;\text{of}\;\text{the}\;\text{analyte}=\frac{\text{HPLC conc}\;(\text{ppb})\times 1\text{mL}\times 100\text{mL}\times 3.3\;(\text{dilution}\;\text{factor})}{4\text{mL}\times \text{Weight}\;\text{of}\;\text{the}\;\text{sample}\;\text{taken}\;(\text{g})}$$

### Derivatization, Detection and Quantification

**Scheme 1:**
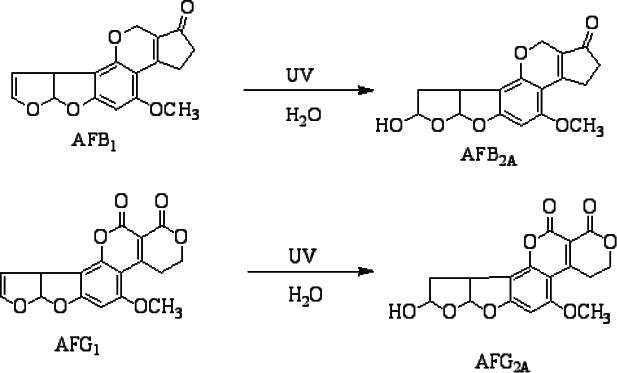
Derivatization of AFB_1_ and AFG_1_

Prior to detection in the HPLC, aflatoxins B_1_ and G_1_ were derivatized into AFB_2A_ and AFG_2A_, respectively, as shown in Scheme 1. The samples were then injected in HPLC coupled with fluorescence detector for determination of aflatoxins. Results were obtained by comparison of peaks' retention times with those of standards of which the peak areas were then used for quantification.

### HPLC Conditions

The eluents were placed in the HPLC for detection of aflatoxins under post-column derivatization with Fluorescence Detector (FLD) (Model Agilent Chem station technology, series 1200). The mobile phase used contained 60:30:10 v/v, water: methanol: acetonitrile. The separations of aflatoxins (AFB_1_, AFB_2_, AFG_1_ and AFG_2_) were performed on the C18 column at a temperature of 30 °C at a flow rate of 1.2 mL/min. The injection volume was 50 µL for both standard solutions and sample extracts. AFG_1_ and AFB_1_ were derivatized to allow their detection in fluorescence detector at an emission wavelength of 465 nm and an excitation wavelength of 360 nm.

### Determination of Percentage Recovery, Limit of Detection and Limit of Quantitation

The accuracy of the procedure was determined by computing the percentage recovery of spiked blank samples. The spices samples were split into two portions, in one portion; a 10 ngmL^-1^ of aflatoxins standards AFB_1_, AFB_2_, AFG_1_ and AFG_2_ was added. Thereafter, the concentration of aflatoxins was determined for both spiked and unspiked samples. The recoveries of all aflatoxins were calculated using equation 4 and the obtained results ranged between 84.3 to 97.1% which was within the acceptable range of 70% to 120%.^23^


4 $$\%\text{Recovery}=\frac{r-b}{s}\times 100\%$$

Where r = the recovered amount, b = blank concentration and s = the spiked amount.

The limit of detection (LOD) and limit of Quantitation (LOQ) of the method were determined as per the method reported by Shrivastara and Gupta (2011).^24^

### Data Analysis

The obtained data were statistically analyzed for identifying whether there was a significant difference between the data obtained from different sampling sites and/or between the different types of spices using MaxStat Lite. Hence, analysis of variance (ANOVA) was applied to relate the mean concentrations of aflatoxins contamination in spices between Zanzibar and Arusha.

## Results

**Figure 3:**
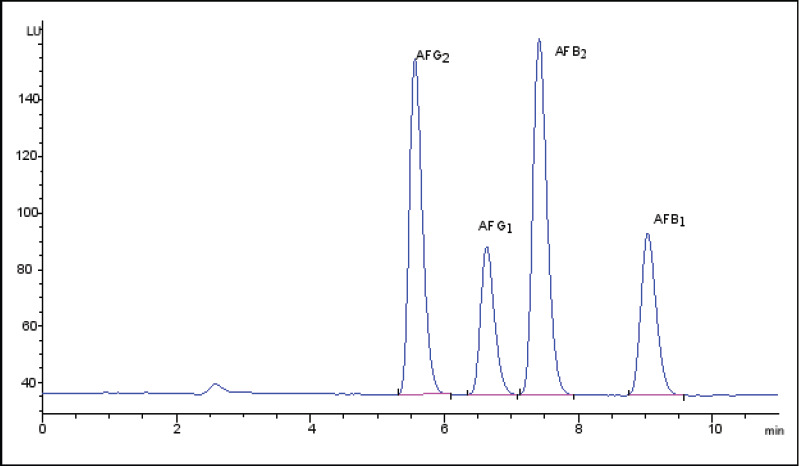
HPLC Chromatogram for Aflatoxins Standard

Qualitative and quantitative determination of aflatoxins was accomplished by running the mixture of aflatoxins standards in HPLC to establish the separation ability of the method. The separation of the four types of aflatoxins was achieved within 10 min. (Figure 3).

For the quantification of aflatoxins, standard solutions of AFB_1_, AFB_2_, AFG_1_ and AFG_2_ were used to obtain the calibration curves at a concentration ranging from 1 to 15 ng/mL. The obtained calibration equations and their respective correlation coefficients are; AFB_1_ (Y = 1.0651x – 0.2616; r^2^ = 0.9983), AFB_2_ (Y = 1.1137x – 0.2836; r^2^ = 0.9982), AFG^1^ (Y = 1.1639x – 0.317; r^2^ = 0.9981), and AFG_2_ (Y = 1.1169x – 0.3032; r^2^ = 0.9982). Table 3 summarizes the results for percentage recovery, limit of detection and limit of quantitation of the method.

**Table 3:** Percentage Recoveries, limit of detection and limit of quantitation of the method

Aflatoxins	% Recovery	LOD (ngmL^-1^)	LOQ (ngmL^-1^)
AFG_2_	88.7	0.13	0.16
AFG_1_	97.1	0.13	0.21
AFB_2_	86.4	0.13	0.18
AFB_1_	84.3	0.16	0.29

### Levels of AFB_1_ AFG_1_, AFG_2_, AFB_2_ and Total Aflatoxins (TAF) in Black Pepper, Garlic, Cardamom and Turmeric

The determined levels of aflatoxins in samples of black pepper, garlic, cardamom and turmeric from Zanzibar and Arusha markets, stores and farms are summarized in Table 4.

**Table 4:** Mean Concentrations of AFG_2_, AFG_1_, AFB_2_, AFB_1_ and TAF for Black Pepper Black Pepper, Garlic, Cardamom and Turmeric Samples Collected from Zanzibar and Arusha Sampling Sites

Spices	Sites		AFG_2_ (ngg^-1^)	AFG_1_ (ngg^-1^)	AFB_2_ (ngg^-1^)	AFB_1_ (ngg^-1^)	TAF (ngg^-1^)

		n	Mean ±SD	Mean ±SD	Mean ±SD	Mean ±SD	Mean ±SD
Black Pepper	DJ	4	0.43±0.29	1.62±0	0.38±0.07	1.56±1.24	0.85±0.45
	MK	4	0.79±0.20	30.26±8.55	0.15±0	1.37±1.09	9.41±5.41
	MKs	3	5.32±0	2.38±1.61	0.73±0.14	6.01±3.34	3.86±1.86
	KZf	2	2.01±0	ND	0.26±0	4.12±0	2.13±0
	KL	4	0.14±0.29	0.33±0	0.13±0	1.87±0	ND
	KLs	3	1.33±0	0.75±0	0.86±0.078	1.20±0.40	1.02±0.25
	SK	3	0.28±0	0.96±1.08	0.16±0	1.98±0.61	1.23±0.53

Garlic	DJ	4	0.21±0	ND	0.18±0	ND	0.22±0
	MK	4	ND	ND	ND	ND	ND
	MKs	3	ND	ND	ND	ND	ND
	KL	4	ND	ND	ND	ND	ND
	KLs	3	0.13±0	ND	0.14±0	ND	ND
	SK	3	ND	ND	ND	ND	ND

Cardamom	DJ	4	0.14±0	ND	0.18±0	ND	0.14±0
	MK	4	2.20±0	ND	ND	19.94±0	11.07±0
	MKs	3	0.76±0.32	ND	ND	0.23±0.21	0.49±0
	KL	4	0.16±0	0.14±0	ND	0.82±0	0.37±0
	KLs	3	ND	0.19±0	0.21±0	1.12±0	0.55±0.52
	SK	3	ND	ND	ND	ND	ND

Turmeric	DJ	4	1.59±0.68	0.21±0.02	0.67±0	2.32±0.88	1.42±0.46
	MK	4	1.76±1.53	ND	0.65±0	1.14±0.79	1.08±0.91
	MKs	3	2.56±0.62	2.71±0	0.61±0	2.41±0.83	2.21±0.48
	KL	4	0.20±0.28	ND	0.30±0	0.35±0.25	0.28±0
	KLs	3	1.61±0.28	0.18±0	ND	ND	0.89±0
	SK	3	0.68±0	ND	0.47±0	3.01±0.61	1.39±0.53

Where n = number of collected samples, ND=Not detected, DJ=Darajani Market, MK=Mwanakwerekwe Market, MKs= Mwanakwerekwe Store, KZf= A spice farm in Zanzibar, KL= Kilombero Market, KLS= Kilombero Stores, SK= Soko Kuu Market

## Discussion

**Black pepper:** The obtained results for total aflatoxins (TAF) in the analyzed spices showed that in black pepper the mean concentration ranged from less than detection limit to 8.41 ngg^-1^. The mean concentrations of TAF were found to vary within sites as indicated in Table 4. High levels of contamination were observed in black pepper samples collected from Mwanakwerekwe market which was 8.41 ± 5.41 ngg^-1^. The observed trend of the mean concentration of TAF in black pepper collected from Zanzibar with respect to sites is Mwanakwerekwe market > Mwanakwerekwe store > Darajani. The observed high levels of total aflatoxins in black peppers collected from Mwanakwerekwe market and stores may be attributed to poor storage conditions which was observed in both market and stores. The use of poor storage facilities like sacks and baskets laid on the floor, might have contributed to the growth of aflatoxins producing fungi due to wetness developed at the bottom part. The spices also stay for long time in stores resulting into increased probability of moisture formation especially at the lower part of the sacks. The observed wet environment of the market/stores caused by human activities such as meat/fish butcheries near spice shops increase the humidity in the surrounding environment and thus favoring growth of aflatoxins producing fungi in the areas around the shops. High temperature in Zanzibar seems to influence the growth of aflatoxins producing fungi. Generally, the individual aflatoxins levels in each market/store were observed to vary from one location to the other; for instance, in Darajani market all four types of aflatoxins were detected though in lower levels. at Mwanakwerekwe market high levels of AFG1 were detected (30.27 ngg^-1^) with relatively low levels of AFG_2_, AFB_1_ and AFB_2_. In Mwanakwerekwe stores; high levels of AFB_1_ were detected (6 ngg^-1^) which exceeded the safe limit set by EC/TBS of 5 ngg^-1^ for AFB_1_.

Black peppers collected from Arusha were observed to have mean concentrations of total aflatoxins ranging from below detection limit (Kilombero market) to 1.23 ± 0.53 ngg-1 (Soko Kuu market). Thus, the general trend of contamination in Arusha was; Soko Kuu market > Kilombero store > Kilombero market. The low levels of aflatoxins in black peppers from Arusha as compared to those from Zanzibar can be attributed to relatively low temperature and humidity as well as good storage conditions.

**Garlic:** The levels of aflatoxins in garlic samples from Mwanakwerekwe market and stores were below detection limit whereas the contamination of samples collected from Darajani market ranged from ND to 0.21 ngg^-1^, 0.18 ngg^-1^ and 0.22 ngg^-1^ for AFG_2_, AFB_2_ and TAF, respectively. The spices with observed contamination were from samples collected from an area very close to meat butcheries and thus, the contamination might be due to the high humid nature of the surrounding environment which favors the growth of aflatoxins producing fungi. Likewise, in the samples collected from Arusha, the levels of aflatoxins were below detection limit in both Soko Kuu market, Kilombero market and samples from the stores. Only samples from Kilombero Stores were observed to have low contamination at levels ranging from ND to 0.13 ngg^-1^ and 0.14 ngg^-1^ of AFG_2_ and AFB_2_, respectively. Low or not detected levels of aflatoxins which was observed in garlic might be attributed to its inhibitory properties towards fungal growth.^25^, ^26^ The findings on garlic are related to results reported in the studies carried out in India by different researchers 27, 28 in which the levels of aflatoxins in garlic were reported to be below detection limit. Similar results were also reported by Abou-Arab et al. (1999), El-Shafie et al. (2002) and Mwangi et al. (2014).^16^, ^25^, ^26^

**Cardamom:** Aflatoxin contamination in cardamom was observed in nearly all market and store samples in Zanzibar. The total aflatoxins contamination levels ranged from 0.14 to 11.07 ngg^-1^. The highest level of AFB_1_ (19.94 ngg^-1^) was observed in samples collected from Mwanakwerekwe market in some shops where cardamom was stored in large baskets and sacks placed on the floor. This increased the chance of the growth of aflatoxins producing fungi due to increased moisture from the lower side of the sacks. The market was observed to be dusty with poor sanitation. Furthermore, the spices were kept in shops for long time before being sold to consumers; this might have contributed to development of fungi in spices. In Mwanakwerekwe stores the levels of AFB_1_ and AFG_2_ ranged from 0.23 to 0.76 ngg^-1^, which was low compared to the levels in market samples. In Darajani market low levels of AFG_2_ and AFB_2_ were detected in some spices samples, however, AFG_1_ and AFB_1_ were not detected.

In Arusha, cardamom collected from Kilombero market had levels ranging from 0.14 to 0.82 ngg^-1^, whereas in Kilombero stores the observed levels were between 0.19 to 1.12 ngg^-1^. No contamination was detected in Soko Kuu market. Lower or not detected levels in samples from Arusha might be due to good handling and storage facilities, and good sanitary environment of both markets and stores. The low temperature and humidity of the region does not favor high growth of the aflatoxins producing fungi. The levels of detected aflatoxins from this study are relatively higher particularly for samples from Mwanakwerekwe market of which the mean TAF concentration was 11.07 ngg^-1^, which exceeded the maximum limit set by TBS/EC compared to other studies which reported low aflatoxins contamination in cardamom due to the presence of cardamom oil.^25^

**Figure 4:**
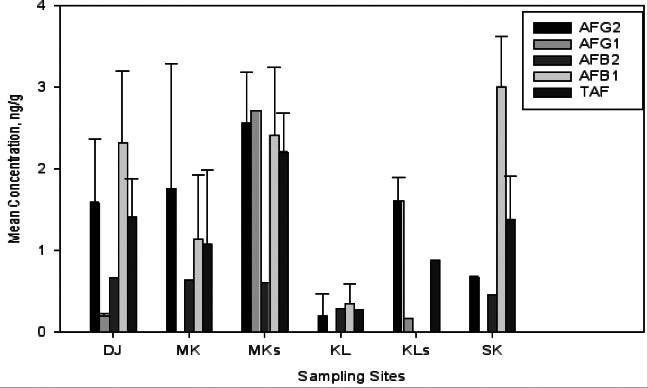
Turmeric Mean Concentration for AFB_1_, AFB_2_, AFG_1_, AFG_2_ and TAF in Zanzibar and Arusha Sampling Sites. Where, DJ = Darajani market, MK = Mwanakwerekwe market, MKs = Mwanakwerekwe store, KL = Kilombero market, KLs = Kilombero Stores, SK = Soko Kuu Market

**Turmeric:** A total of 16 (72%) samples out of 22 turmeric samples were found to be contaminated with aflatoxins from both markets and stores but the levels did not exceed the limits set by EC/TBS. Samples of turmeric from Darajani market in Zanzibar had levels of contamination ranging from 0.21 to 2.32 ngg^-1^. At Mwanakwerekwe stores and market some spices were contaminated, which might be due to improper storage of the spices that might have led to random movement of dust containing aflatoxins fungal spores from one point to another. Turmeric samples collected from Arusha had mean concentration of TAF ranging from 0.28 to 2.21 ngg^-1^ (Figure 4). High levels of AFB1 (3.01 ± 0.61 ngg^-1^) were found in Soko Kuu market samples. The shop at which samples were taken was located nearby water ways and washrooms hence the wetness in the surrounding environment might have contributed to the growth of producing fungi. On the other hand, low levels were observed in both Kilombero market and stores. The turmeric spices in these locations were stored in good storage facilities that do not encourage fungal growth.

### Comparison of the levels of Aflatoxins between Spices from Zanzibar and Arusha

**Figure 6:**
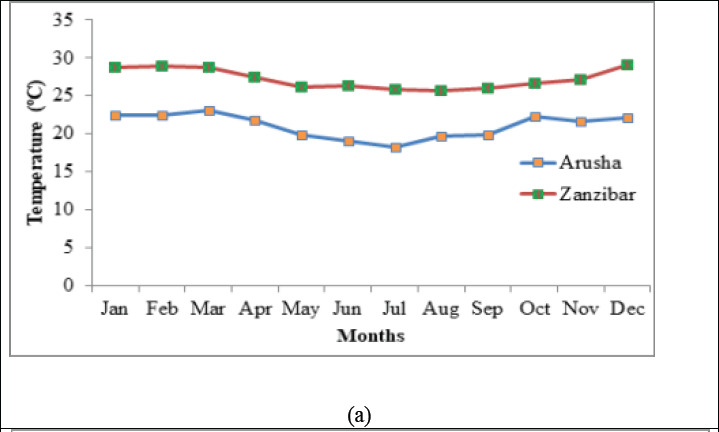

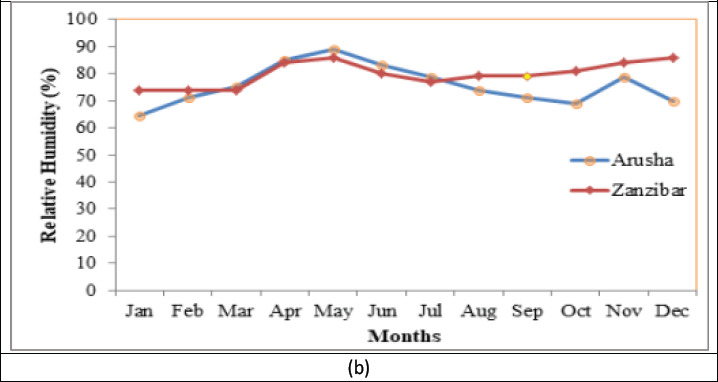
(a) Annual mean monthly temperature for Zanzibar and Arusha, (b) Annual Relative Humidity for Zanzibar and Arusha

The observation made in this study revealed that, the mean concentrations of total aflatoxins contamination in black pepper samples from Zanzibar was higher (4.71 ngg^-1^) than for the same samples from Arusha (1.12 ngg^1^). The higher mean concentration of TAF in black pepper samples from Zanzibar might be attributed to high temperature and humidity observed in Zanzibar as well as poor storage facilities and storage condition in the markets and stores where spices are stored in sacks and buckets on the floor. Again, the shops are surrounded by meat butcheries which contributes wetness in the surrounding environments. The levels of TAF in turmeric from Zanzibar was slightly higher (1.82 ngg^-1^) than that detected in samples from Arusha which was 1.03 ngg^-1^. For garlic the mean concentration of TAF from Zanzibar was somehow high (0.22 ngg^-1^) in comparison to that of Arusha which was 0.11 ngg^-1^. Generally, garlic samples had very low levelsf aflatoxins compared to other studied spices. The study also revealed that the mean concentration of TAF in cardamom from Zanzibar was higher (0.37 ngg^-1^) than that detected in samples from Arusha which was 0.21 ngg^-1^. It was also observed that the contamination in samples collected from Zanzibar is a result of long storage time of spices in shelves before reaching the final consumers. Furthermore, Figure 6 shows the comparison of the mean concentration of TAF in spices from Zanzibar and Arusha.

Furthermore, the observed high levels of aflatoxins contamination in spices collected from Zanzibar sites can be attributed to the high temperature and high humidity of the area. During the sampling period, the temperature ranged from 25.4 to 29.8 °C and RH from 77 to 90% as was recorded by Zanzibar Meteological Agency (ZMA). For the case of Arusha, the observed low levels of aflatoxins contamination in spices collected from Soko Kuu and Kilombero market is attributed to the low temperature and humidity as it was recorded by Tanzania Meteological Agency (TMA) - Arusha Centre. The temperature during the sampling period ranged from 21.6 to 22.8 °C and RH from 70.8 to 78.4% (Figure, 6a and b). The temperature which favors fungal growth ranges from 25 to 40 °C.^29^. Toxin production decreases or not produced at low temperature, unless the ambient relative humidity (RH) increases. RH that favors growth of Aflatoxins producing fungi ranges from 80% to 99%.^29^

## Conclusion

In Tanzania, many spices such as ginger, cardamom, cinnamon, turmeric, black pepper, nutmeg, vanilla, chili, cumin, onion, garlic and cloves are cultivated and due to the existence of conditions favorable for the growth of aflatoxins producing fungi, they are prone to contamination with aflatoxins at different levels. The investigated four types of spices; cardamom, turmeric, garlic and black pepper which are produced and/or marketed in Zanzibar and Arusha have indicated the necessity of quick intervention to raise awareness to not only spices users but also sellers for proper handling and storage. A total of 7 (8.33 %) samples out of all analyzed spice samples were contaminated with Aflatoxin B_1_ that exceeded the set limits of 5 ngg^-1^ set by EC/TBS. Generally, the obtained results indicate a risk to public health and hence gives an alarm for intervention measures.

## Figures and Tables

**Figure 5:**
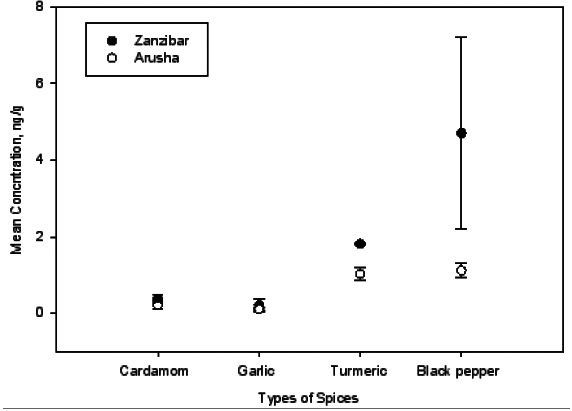
Mean Concentration for TAF in Zanzibar and Arusha Spices

## References

[1] Shephard GS (2003). Aflatoxin and food safety: recent African perspectives. Journal of Toxicology: Toxin Reviews.

[2] Williams JH, Phillips TD, Jolly PE, Stiles JK, Jolly CM, Aggarwal D (2004). Human aflatoxicosis in developing countries: a review of toxicology, exposure, potential health consequences, and interventions. The American Journal of Clinical Nutrition.

[3] Jackson LS, Al-Taher F, Elsevier (2008). Factors affecting mycotoxin production in fruits.

[4] Tosun H, Arslan R (2013). Determination of aflatoxin B1 levels in organic spices and herbs. The Scientific World Journal.

[5] Peraica M, Radić B, Lucić A, Pavlović M (1999). Toxic effects of mycotoxins in humans. Bulletin of the World Health Organization.

[6] Wild CP, Montesano R (2009). A model of interaction: aflatoxins and hepatitis viruses in liver cancer aetiology and prevention. Cancer Letters.

[7] Wu F, Khlangwiset P (2010). Health economic impacts and cost-effectiveness of aflatoxin-reduction strategies in Africa: case studies in biocontrol and post-harvest interventions. Food Additives and Contaminants.

[8] Bennett JW, Klich M (2003). Mycotoxins. Clinical Microbiology Reviews.

[9] Chiavaro E, Dall'Asta C, Galaverna G, Biancardi A, Gambarelli E, Dossena A, Marchelli R (2001). New reversed-phase liquid chromatographic method to detect aflatoxins in food and feed with cyclodextrins as fluorescence enhancers added to the eluent. Journal of Chromatography. A.

[10] Brera C, Caputi R, Miraglia M, Iavicoli I, Salerno A, Carelli G (2002). Exposure assessment to mycotoxins in workplaces: aflatoxins and ochratoxin A occurrence in airborne dusts and human sera. Microchemical Journal.

[11] Colak H, Bingol EB, Hampikyan H, Nazli B (2006). Determination of aflatoxin contamination in red-scaled, red and black pepper by ELISA and HPLC. Journal of Food and Drug Analysis.

[12] Jalili M (2016). Natural occurrence of aflatoxins contamination in commercial spices in Iran. Iranian Journal of Health, Safety and Environment.

[13] Hashem M, Alamri S (2010). Contamination of common spices in Saudi Arabia markets with potential mycotoxin-producing fungi. Saudi Journal of Biological Sciences.

[14] Fufa H, Urga K (1996). Screening of aflatoxins in Shiro and ground red pepper in Addis Ababa. Ethiopian Medical Journal.

[15] Rajarajan P, Rajasekaran K, Devi NA (2013). Aflatoxin contamination in agricultural commodities. Indian Journal of Pharmaceutical and Biological Research.

[16] Mwangi WW, Nguta CM, Muriuki BG (2014). Aflatoxin contamination in selected spice preparations in the Nyahururu retail market, Kenya. Journal of Scientific Research and Reports.

[17] Kimanya ME, De Meulenaer B, Tiisekwa B, Ndomondo-Sigonda M, Devlieghere F, Van Camp J, Kolsteren P (2008). Co-occurrence of fumonisins with aflatoxins in home-stored maize for human consumption in rural villages of Tanzania. Food Additives and Contaminants.

[18] Kimanya ME, Shirima CP, Magoha H, Shewiyo DH, De Meulenaer B, Kolsteren P, Gong YY (2014). Co-exposures of aflatoxins with deoxynivalenol and fumonisins from maize based complementary foods in Rombo, Northern Tanzania. Food Control.

[19] Geary PA, Chen G, Kimanya ME, Shirima CP, Oplatowska-Stachowiak M, Elliott CT, Routledge MN, Gong YY (2016). Determination of multi-mycotoxin occurrence in maize based porridges from selected regions of Tanzania by liquid chromatography tandem mass spectrometry (LC-MS/MS), a longitudinal study. Food Control.

[20] Magembe K, Mwatawala M, Mamiro D, Chingonikaya E (2016). Assessment of awareness of mycotoxins infections in stored maize (Zea mays L.) and groundnut (arachis hypogea L.) in Kilosa District, Tanzania. International Journal of Food Contamination.

[21] Mohammed S, Munissi JJE, Nyandoro SS (2018). Aflatoxin levels in sunflower seeds and unrefined sunflower oil from Singida, Tanzania. Food Additives and Contaminants: Part B.

[22] Boni SB, Beed F, Kimanya ME, Koyano E, Mponda O, Mamiro D, Kaoneka B, Bandyopadhyay R, Korie S, Mahuku G (2021). Aflatoxin contamination in Tanzania: quantifying the problem in maize and groundnuts from rural households. World Mycotoxin Journal.

[23] Shah VP, Midha KK, Findlay JW, Hill HM, Hulse JD, McGilveray IJ, McKay G, Miller KJ, Patnaik RN, Powell ML (2000). Bioanalytical method validation—a revisit with a decade of progress. Pharmaceutical Research.

[24] Shrivastava A, Gupta VB (2011). Methods for the determination of limit of detection and limit of quantitation of the analytical methods. Chronicles of Young Scientists.

[25] Elshafie AE, Al-Rashdi TA, Al-Bahry SN, Bakheit CS (2002). Fungi and aflatoxins associated with spices in the Sultanate of Oman. Mycopathologia.

[26] Abou-Arab A, Kawther MS, El Tantawy M, Badeaa RI, Khayria N (1999). Quantity estimation of some contaminants in commonly used medicinal plants in the Egyptian market. Food Chemistry.

[27] Hammami W, Fiori S, Al Thani R, Kali NA, Balmas V, Migheli Q, Jaoua S (2014). Fungal and aflatoxin contamination of marketed spices. Food Control.

[28] Reddy K, Reddy C, Muralidharan K (2009). Potential of botanicals and biocontrol agents on growth and aflatoxin production by Aspergillus flavus infecting rice grain. Food Control.

[29] Milani JM (2013). Ecological conditions affecting mycotoxin production in cereals: a review. Veterinarni Medicina.

